# The impact of hypothetical interventions on adiposity in adolescence

**DOI:** 10.1038/s41598-021-90415-z

**Published:** 2021-05-27

**Authors:** Mekdes K. Gebremariam, Roch A. Nianogo, Nanna Lien, Mona Bjelland, Knut-Inge Klepp, Ingunn H. Bergh, Yngvar Ommundsen, Onyebuchi A. Arah

**Affiliations:** 1 Department of Nutrition, Faculty of Medicine, University of Oslo, Oslo, Norway; 2 Department of Epidemiology, Fielding School of Public Health, University of California, Los Angeles (UCLA), Los Angeles, CA USA; 3 Division of Mental and Physical Health, Norwegian Institute of Public Health, Oslo, Norway; 4 Department of Health and Inequality, Norwegian Institute of Public Health, Oslo, Norway; 5 Department of Coaching and Psychology, Norwegian School of Sport Sciences, Oslo, Norway; 6 Department of Statistics, UCLA College of Letters and Science, Los Angeles, CA USA; 7 California Center for Population Research, UCLA, Los Angeles, CA USA

**Keywords:** Medical research, Risk factors

## Abstract

In order to develop effective public health initiatives aimed at promoting healthy weight development, identifying the interventions/combination of interventions with the highest beneficial effect on body weight is vital. The study aimed to estimate the mean BMI at age 13 under hypothetical interventions targeting dietary behavior, physical activity and screen time at age 11. We used data from a school-based cohort study of 530 participants followed between the ages of 11 and 13. We used g-computation, a causal modeling method, to estimate the impact of single and combined hypothetical behavioral interventions at age 11 on BMI at age 13. Of the hypothetical interventions, the one with the largest population mean difference in BMI was the one combining all interventions (dietary behavior, physical activity and screen time interventions) and assuming 100% intervention adherence, with a population mean differences of − 0.28 (95% CI − 0.59, 0.07). Isolated behavioral interventions had a limited impact on BMI. This study demonstrated that a combination of healthy dietary behavior and physical activity promotion, as well as screen time reduction interventions at age 11 could have the highest beneficial effect on the reduction of BMI at age 13, although the change in BMI was small. The findings highlight the importance of a systems approach to obesity prevention focusing on multicomponent interventions.

## Introduction

The obesity epidemic remains a significant public health challenge globally. Although stabilization and even some reversal in the rates of overweight (OW) and obesity (OB) have been observed in several high-income countries in recent years, a substantial proportion of children and adolescents remain affected by the epidemic^1,2^. Children and adolescents with OW and OB are at risk of short- and long-term consequences of excessive body fat^3–7^, including the tracking of body weight into adulthood^8^.

Extensive obesity prevention efforts ranging from individual interventions to environmental/ policy changes have been implemented over the past decades. Although favorable intervention effects have been documented, results have not always been uniform or clear^9–14^. The development of effective obesity prevention interventions thus remains a public health priority. Evidence to inform such interventions would ideally be generated from randomized controlled trials. However, problems of external validity might exist as participants in such studies might not always be representative of the real-world population that the interventions might eventually target. More importantly, such trials are often difficult to conduct because of different challenges, both practical (e.g. difficulty to reach everyone in the target group, costs related to implementing interventions, difficulty of achieving sustained adherence to interventions among children) and ethical (e.g. not exposing participants to beneficial interventions). A useful alternative or supplemental approach is the use of causal modeling methods applied to observational data, in order to estimate the potential effects of hypothetical interventions on pre-defined outcomes under realistic or real-world scenarios. Such modern modeling methods include the use of the g-computation algorithm to assess the potential effects of single or combined interventions on body weight^15^. By applying this approach, it is possible to quantify the population impact of a particular health intervention on childhood obesity when all children are exposed to the intervention. It is also possible to explore the interventions or combination of interventions with the highest impact on body weight. Such findings would provide useful information for policy makers aiming to implement successful obesity prevention interventions. G-computation has previously been applied to assess the impact of hypothetical interventions related to body weight^16,17^ as well as non-communicable^18,19^ and communicable^20^ diseases, mainly among adults.

The overall aim of this paper was thus to estimate the impact of hypothetical behavioral interventions (separately and combined, with different adherence levels) at age 11 on the BMI of 13 year-olds, by applying g-computation (using the parametric g-formula) to longitudinal data.

## Methods

### Design and sample

The participants of the HEIA cohort study were students from 25 control schools of the HEalth In Adolescents (HEIA) study^21^. Schools were included in this study if they had a minimum of 40 enrolled students in the 6th grade. Schools were thus recruited from the largest towns/municipalities in seven counties from the Eastern part of Norway. Twenty-five of the schools were randomly assigned to the control group and twelve to the intervention group.

All 6th graders in the 25 control schools included in this study and their parents/legal guardians were invited to participate in the baseline study which took place in September 2007. Parental consent was obtained for 73% (n = 1014) of children from these schools. A total of 975 students (96% of the 1014 returning parental informed consent) from these schools participated at baseline. A follow-up was conducted in May 2009. A total of 908 students participated at both time points.

The study was approved by the Regional Committees for Medical Research Ethics and the Norwegian Social Science Data Service. The research was carried out in accordance with relevant ethical guidelines and regulations. All parents of participating adolescents provided written informed consent. The adolescents themselves provided assent for participation. Anonymity and confidentiality were ensured.

### Data collection and measures

The adolescents answered an internet-based questionnaire, when necessary assisted by research assistants. Anthropometric measurements were conducted by research group members. Physical activity (PA) was measured using accelerometers.

#### Outcome variable

Weight and height were objectively measured by same sex project staff at baseline and at follow-up. The adolescents were measured in light clothing and bare foot. Body mass index (BMI) was calculated as weight/height^2^. The BMI at follow-up was used as the outcome in this study.

#### Exposure/intervention variables

PA was assessed using accelerometers (ActiGraph GT1M/model 7164, LLC, Pensacola, FL, USA). The adolescents were instructed to wear accelerometers for 5 consecutive days, during all waking hours, except when doing water activities (as monitors were not waterproof). Moderate- to-vigorous PA (MVPA) was defined as all activity at intensities above 2000 counts per minute^22^. MVPA was expressed as min/day of accelerometer activity measured. Details of the instructions provided to students and of the data handling and extraction procedures are available elsewhere^23^.

Four questions with pre-coded answer categories were asked to assess usual TV/DVD use and use of computer/electronic games: How many hours do you usually watch TV and/or DVD on a normal weekday? The same question was asked for a normal weekend day. The answer categories were (recoding in brackets): half hour [0.5], 1 h [1], 2 h [2], 3 h [3], 4 h [4], 5 h or more [5]. The two questions on computer/ electronic game use were formulated in the same way as for TV/DVD, but the answer categories were: no playing [0], half hour or less [0.5], 1 h [1], 2 h [2], 3 h [3], 4 h or more [4]. Weekly scores for TV/DVD and computer/electronic games were calculated by summing hours reported for an average weekday (multiplied by 5) and average weekend day (multiplied by 2).

The consumption of fruit and vegetables (raw and cooked) was assessed by three frequency questions with eight answer categories ranging from never/seldom to three times per day or more. The questions assessing the intake of fruits and vegetables have been validated among 11-year-olds with a 7-day food record as the reference method. They were found to have a satisfactory ability to rank subjects according to their intake of fruits and vegetables^24^. The frequency of consumption of unhealthy snacks (candies, chocolate, sweet biscuits, buns/muffins and salty snacks (e.g. potato chips)) was assessed using questions with seven answer categories ranging from never/seldom to two times per day or more. The intake of sugar-sweetened soft drinks (sum of sugar-sweetened carbonated soft drinks and fruit drinks) during weekdays was assessed using two frequency questions (with categories ranging from never/seldom to every weekday); amount was assessed in glass (from one to four or more, with one glass = 1.67 deciliters). Total intake during weekdays was assessed by multiplying frequency of intake by amount of intake. The adolescents were also asked about the total amount of sugar-sweetened soft drinks consumed in the weekends using two questions with categories ranging from never/seldom to 7 glasses or more. Weekday and weekend intake values were summed up to create a weekly intake variable. The questions assessing the intake of sugar-sweetened soft drinks have been validated among 9- and 13-year-olds using a 4-day pre-coded food diary as the reference method, although separate assessment of weekday and weekend intakes was not done in that study. Moderate correlation coefficients were obtained^25^.

Breakfast intake was assessed using the question: “How often do you eat breakfast?” with nine response categories ranging from “never” to “every day”. Breakfast consumption was categorized into two: those consuming breakfast every day vs. others, because the continuous variable was highly skewed and because the recommendation for breakfast intake is to not skip breakfast.

The questions assessing the use of TV/DVD and the use of computer/electronic games have shown evidence of adequate test–retest reliability in a separate study conducted prior to the main survey^21^. The same was true for the measures assessing the intake of fruits, vegetables and soft drinks^21^.

#### Confounders

Age (in years), sex, parental education, pubertal development, and baseline BMI were included as confounders.

Parental education was reported by parents as part of the parental informed consent for the adolescent. It was categorized into ‘low’ (12 years or less), ‘medium’ (between 13 and 16 years) and ‘high’ (more than 16 years). Educational status of the parent with the longest education (or else the one available) was used in the analyses.

The Pubertal Development Scale (PDS), based on the pubertal category scores defined by Carskadon and Acebo^26^, was used to assess pubertal status. The students self-reported this information on a separate paper questionnaire. PDS for boys included body hair growth, voice, and facial hair. For girls, PDS included body hair growth, breast development, and menarche. The categories were: pre-pubertal, early pubertal, mid-pubertal, late pubertal or post-pubertal. For the analyses, dichotomization into pre/early pubertal and mid/late/post-pubertal was made for girls and into pre-pubertal and early/mid/late/post-pubertal for boys. Such categorization was suggested by a study that compared the PDS and the Sexual Maturation Scale^27^.

### Statistical analysis

The g-computation algorithm (applied using the parametric g-formula) was used to predict the potential mean BMI at age 13 under different hypothetical intervention scenarios. Compared to conventional methods, g-computation has the advantages of handling joint and dynamic interventions, allowing for the estimation of multiple (causal) parameters and yielding population average estimates^18,28,29^. First, linear regression models of the outcome BMI on the risk factors were fit, adjusting for age, puberty, sex, parental education and BMI at age 11. Second, the regression coefficients obtained from these models were used to predict the potential outcomes under the different hypothetical interventions (individual and combined). Third, the “intervention impact” was obtained by calculating the difference between the predicted potential mean BMI under the various scenarios (100% of the population exposed to the desired level of the risk factor) and the BMI under no intervention (exposure distributions equal to that of the original sample). Fourth, standard errors and 95% confidence intervals were obtained via bootstrapping. In addition, scenarios under which only 25%, 50% and 75% of the population would adhere to the interventions were also used to predict mean BMI at age 13. These different levels across the adherence spectrum were chosen since actual adherence levels to different behavioral interventions can vary. The following assumptions were made: no uncontrolled confounding, positivity, consistency and no other source of bias^30^. Clustering effect at the school level was checked. It was found that only 1.5% of the total variation in BMI was at the school level. Thus, adjustment for clustering effect was not done. Analyses were also conducted to explore whether those with missing data and those who did not participate at both time points differed substantially from their counterparts.

### Hypothetical interventions

In this study, we assessed the impact of intervening on late childhood behavioral risk factors on early adolescence obesity. To do so, we identified relevant interventions that have the potential to change BMI later in adolescence. These interventions were based on available recommendations for behaviors such as PA, fruits and vegetable consumption, etc. For example, as the World Health Organization recommends children engage in MVPA for at least 60 min each day^31^, we can design a “hypothetical” intervention that will essentially move students from exercising less than 60 min/day MVPA to 60 min/day MVPA or more. Assuming a 100% uptake of the intervention, children who are not yet physically active will increase their PA level under the intervention. Similarly, children already meeting the recommendation of 60 min/day MVPA will remain in this category under the intervention. All other interventions were created using a similar reasoning: all children meet the recommendations for the intake of fruits and vegetables (5 per day), the recommendations for breakfast intake (daily intake of breakfast), the recommendations for daily screen time (less than 2 h per day) and do not consume sugar-sweetened beverages. In addition, we assessed: (i). the combined effects of all dietary interventions, (ii). the effects of PA and screen time interventions combined and (iii) the effects of all dietary, PA and screen time interventions combined, on early adolescence BMI. In addition to the hypothetical scenario which assumes 100% adherence to interventions, we also simulated different scenarios whereby intervention adherence was set to 25%, 50% and 75%.

## Results

Among the adolescents who participated in the study at both time points, 248 did not have valid accelerometer data. An additional 130 participants were excluded due to missing data on different variables. The final analytic sample thus consisted of 530 adolescents with complete data on all variables used in the analyses.

Table 1 shows the characteristics of participants. The mean age of participants at baseline was 11.2 and 52% of the participants were female. The mean BMI at baseline was 17.8 kg/m^2^. Twelve percent of the adolescents were OW/OB at baseline. Sixty four percent met the daily PA recommendation of 60 min per day MVPA while 42% met the recommendations of 2 h or less per day of screen time. Only 18% met recommendations for fruit and vegetable consumption (5 per day). Ninety percent ate breakfast on a daily basis, as recommended. Sixteen percent reported not consuming sugar-sweetened beverages. There was no substantive difference between those who participated at both time points and those who did not in terms of the different variables of interest in this study. Those with missing data did similarly not differ from those with complete data. The only exception was for sex, whereby boys were more likely to have missing data compared to girls (p = 0.003).

**Table 1:** Baseline and follow-up characteristics of participants (n = 530).

Characteristics	Mean (SD) or Percentage
Mean baseline age in years (SD)	11.2 (0.2)
Boys	48%
Parental education (% low)	34%
Meets the daily 60 min PA recommendation	64%
Meets the daily 5 fruits and vegetables recommendation	18%
Meets the daily 0 limit SSB recommendation	16%
Meets the daily consumption of breakfast recommendation	90%
Meets the daily less than 2 h screen time recommendation	42%
Mean baseline BMI (SD)	17.8 (2.5)
Baseline overweight/obese	12%
Mean follow-up BMI (SD)	18.8 (2.7)

*BMI* body mass index, *PA* physical activity, *SSB* sugar-sweetened beverages.

**Figure 1:**
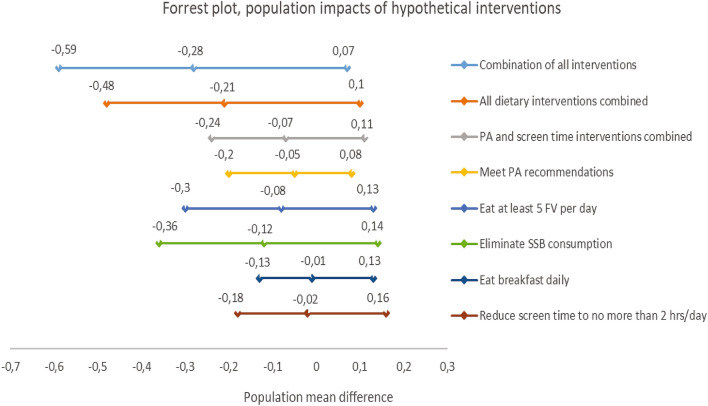
Impacts of hypothetical behavioral interventions at age 11 on BMI at age 13. *FV* fruits and vegetables, *PA* physical activity, *SSB* sugar-sweetened beverages. Adjusted for age, puberty, sex, parental education, BMI at age 11.

The mean BMI at follow-up under no intervention was 18.8 (95% CI 18.6–19.04) and 18.5 (95% CI 18.2–18.9) with all interventions combined, when 100% adherence to the interventions was assumed. Of the simulated interventions (100% adherence), the one with the largest population mean difference in BMI was the one combining all interventions (dietary and PA interventions), with a population mean difference of − 0.28 (95% CI − 0.59, 0.07). The intervention with the second highest mean difference was that combining all dietary interventions with a population mean difference of − 0.21 (95%CI − 0.48, 0.10) (Fig. 1, Table 2).

**Table 2:** Mean BMI at age 13 under interventions at age 11 and impact of different interventions in terms of mean differences (n = 530).

Interventions (100% adherence)	Mean BMI	Mean BMI (lower bound)	Mean BMI (Upper bound)	MD (Point estimate)	MD (lower bound)	MD (upper bound)
No intervention, natural course	18.81	18.60	19.04			
Increase PA from less than 60 min/day to more than 60 min/day	18.76	18.52	19.00	− 0.05	− 0.20	0.08
Increase FV consumption from less than 5/day to more than 5/day	18.73	18.47	19.02	− 0.08	− 0.30	0.13
Reduce SSB consumption from more than 0 times/day to 0 times/day	18.69	18.39	19.04	− 0.12	− 0.36	0.14
Increase breakfast consumption from none/day to once/day	18.80	18.57	19.06	− 0.01	− 0.13	0.13
Reduce screen time from more than 2 h/day to less than 2 h/day	18.79	18.52	19.06	− 0.02	− 0.18	0.16
Combination of all dietary intervention: up FV, up breakfast, down SSB	18.60	18.23	19.01	− 0.21	− 0.48	0.10
Combination of all PA interventions: up PA, down screen time	18.74	18.49	18.99	− 0.07	− 0.24	0.11
Combination of all interventions	18.53	18.17	18.90	− 0.28	− 0.59	0.07

Using 200 bootstrap samples, marginal structural model.

Outcome of interest = BMI at age 13.

Adjusted for age, puberty, sex, parental education, BMI at age 11.

*BMI* body mass index, *FV* fruits and vegetables, *MD* mean difference, *PA* physical activity, *SSB* sugar-sweetened beverages.

The population mean difference in BMI for all interventions combined and with adherence levels of 25%, 50% and 75% was − 0.07 (− 0.14, 0.01), − 0.14 (− 0.27, 0) and − 0.20 (− 0.40, 0.01) respectively (Table 3).

**Table 3:** Mean BMI at age 13 under interventions at age 11 and impact of interventions at different adherence levels in terms of mean differences.

Interventions	25% adherence		50% adherence		75% adherence	
	Mean BMI (CI)	MD	Mean BMI (CI)	MD	Mean BMI (CI)	MD
Increase PA from less than 60 min/day to more than 60 min/day	18.78 (18.57, 19.01)	− 0.01 (− 0.04, 0.01)	18.78 (18.55, 19.00)	− 0.03 (− 0.07, 0.01)	18.76 (18.53, 18.99)	− 0.04 (− 0.10, 0.02)
Increase FV consumption from less than 5/day to more than 5/day	18.78 (18.55, 19.01)	− 0.02 (− 0.06, 0.02)	18.76 (18.49, 19.01)	− 0.04 (− 0.13, 0.05)	18.74 (18.46, 19.01)	− 0.06 (− 0.19, 0.07)
Reduce SSB consumption from more than 0 times/day to 0 times/day	18.77 (18.54, 19.01)	− 0.03 (− 0.08, 0.03)	18.74 (18.49, 19.00)	− 0.06 (− 0.15, 0.05)	18.71 (18.45, 18.99)	− 0.08 (− 0.24, 0.08)
Increase breakfast consumption from none/day to once/day	18.79 (18.57, 19.03)	0.00 (− 0.01, 0.01)	18.79 (18.56, 19.03)	0.00 (− 0.02, 0.01)	18.80 (18.58, 19.03)	0.00 (− 0.03, 0.02)
Reduce screen time from more than 2 h/day to less than 2 h/day	18.79 (18.57, 19.02)	− 0.01(− 0.04, 0.02)	18.79 (18.56, 19.03)	− 0.01 (− 0.07, 0.05)	18.78 (18.53, 19.03)	− 0.02 (− 0.11, 0.07)
Combination of all dietary intervention: up FV, up breakfast, down SSB	18.75 (18.51, 18.99)	− 0.05(− 0.11, 0.02)	18.70 (18.44, 18.98)	− 0.10 (− 0.23, 0.03)	18.65 (18.36, 18.95)	− 0.15 (− 0.34, 0.06)
Combination of all PA interventions: up PA, down screen time	18.78 (18.55, 19.01)	− 0.02 (− 0.05, 0.01)	18.76 (18.52, 19.00)	− 0.04 (− 0.10, 0.03)	18.74 (18.48, 19.00)	− 0.06 (− 0.15, 0.04)
Combination of all interventions	18.73 (18.49, 18.96)	− 0.07 (− 0.14, 0.01)	18.66 (18.39, 18.93)	− 0.14 (− 0.27, 00)	18.59 (18.27, 18.91)	− 0.20 (− 0.40, 0.01)

Using 2000 bootstrap samples, marginal structural model.

Outcome of interest = BMI at age 13.

Adjusted for age, puberty, sex, parental education, BMI at age 11.

*BMI* body mass index, *FV* fruits and vegetables, *MD* mean difference, *PA* physical activity, *SSB* sugar-sweetened beverages.

## Discussion

This study aimed to assess the impact of hypothetical behavioral interventions (individual and joint) at age 11 on BMI at age 13 using data from a cohort study analyzed using g-computation. We found that a combination of dietary, PA and screen time interventions at age 11 led to the largest population mean difference in BMI at age 13, using different adherence levels to interventions. Findings also indicated that combined dietary interventions may result in favorable changes in BMI. Interventions at age 11 targeting PA and screen time only appeared to have a limited effect on population level BMI at age 13.

Assessing the population impact of interventions or combination of interventions on the development of OW/OB under the scenario where every child is exposed to these interventions is important for informing policy and practice. The development of OW and OB is the result of complex interacting factors. An imbalance of energy intake and energy output is at the core of this complexity. It is thus vital to understand which interventions/combinations of interventions targeting energy balance-related behaviors would result in the most relevant changes in population level BMI. In line with the findings of this study, previous interventions have indicated that a combination of PA and dietary behavior interventions have the highest impact on BMI among children^9,10^. These findings support the need to adopt an approach to obesity prevention focusing on multicomponent interventions. The difference in BMI documented in this study after exposure to the interventions is small (even with 100% adherence), but can lead to clinically relevant shift in population BMI if maintained over time. Similar to these findings, meta-analyses of obesity-related interventions document rather small/modest impacts of interventions^9,10,12,13^, although the reach of and the adherence to these interventions, which is often not assessed, could also impact these results.

The single behavior intervention with the highest influence on population level BMI was the one targeting the intake of sugar-sweetened beverages, which is in line with evidence from systematic reviews supporting the impact of sugar-sweetened beverages on the development of OW/OB among youth^32,33^. Increasing fruit and vegetable intake to five times or more per day by itself had a limited impact on population level BMI. A systematic review concluded that it remains unclear whether fruit and vegetable intake in isolation from lower caloric intake or increased PA will have an impact on adiposity^34^. A combination of dietary behavior interventions was however found to result in favorable changes in BMI, reflecting the additive impact of different dietary behaviors on body weight.

PA interventions did not have a substantive effect on BMI, a finding also reported in meta-analyses of PA interventions among children^11,35^, and in a review of prospective studies^36^ but in contrast to other reviews^12,13^. The study found that interventions to limit screen time to less than 2 h per day had a limited impact on the BMI of the adolescents. A systematic review of reviews suggested that cross-sectional studies reported small associations between screen time and adiposity but that associations documented in longitudinal studies were less consistent^37^. The cut-off of 2 h per day often recommended to define unhealthy screen time is however also not specific for impacts of screen time on obesity. More evidence is needed in this regard to define a cut-off that might be more clinically relevant for the impact of sedentary behavior on adiposity^38^. The results of this study also showed the importance of a high adherence to interventions to achieve a substantial population level effect on BMI.

However, it is also important to take into consideration that the percentage of the population that needs to be exposed to the different interventions also affects the size of the mean population difference in BMI following the interventions. For example, for interventions such as the elimination of soft drink consumption, a high percentage of the population needs to be exposed to the intervention, whereas for breakfast intake only a small percentage of the population needs to “receive” the intervention.

Although some of the behavioral interventions investigated in this study did not show an impact on BMI, it should be recognized that the behaviors included have other beneficial health effects. Thus, these behaviors should still be targeted in future interventions.

### Strengths and weaknesses

The present study has several strengths including the use of objective anthropometric measurements, the objective measurement of PA and sedentary time and the inclusion of several energy balance-related behaviors. The application of a causal inference method to the data is also a strength of the study. Given the well-known challenges in conducting rigorous randomized controlled trials, the results of this study provide useful information that can inform policy and practice in this area.

However, the study should be seen in light of the following weaknesses. Using self-report measures of dietary behaviors is associated with issues of reliability and validity. However, several measures used in this study had evidence of test–retest reliability and validity as indicated. A more complete assessment of energy intake would have provided more useful findings as only selected dietary behaviors were included in this study. There was missing data, namely due to the exclusion of participants who lacked valid accelerometer data; there was also some loss to follow-up. However, those with missing data did not differ from those with complete data, with the exception of sex differences. The twenty month period between age 11 and 13 might also be relatively short in order to detect substantial changes in BMI.

Like for any observational estimates, the validity of our results rely on the assumptions made. First, there is an untestable assumption of exchangeability^30^ between those who are exposed to a certain behavior and those who are not. In fact, the projections are based on association estimates between the various exposures and adiposity which are assumed to be causal given sufficient covariate adjustment to remove confounding. This assumption is also known as the no uncontrolled confounding assumption or the ignorable treatment assignment assumption given adjusted covariates. In this study we controlled for important potential confounders. However, energy balance-related behaviors and body weight are complex factors with multilevel influences. Thus, totally accounting for confounders can be challenging. Second, this study also makes the so-called consistency assumption^30,39^; that is, for each subject, it assumes that the potential outcome under the observed value of the exposure is equal to the observed outcome. Said differently, a child’s BMI under a hypothetical intervention, say, a physical activity (PA) intervention, for a child who is, in fact, observed to be physically active (i.e., meet the PA recommendation) should not be different than the child’s actual BMI. The assumption holds by expectation at the population level. If this assumption is met, then the interventions as defined in this study would be considered sufficiently well-defined interventions. We believe that the interventions here are sufficiently well-defined because they focus on changing a particular behavior. Third, we made the positivity assumption^40^, which requires that both treated and untreated subjects exist at each level of the confounders; that is, there is no deterministic uptake of exposure by individuals with a probability 0 or 1 in each subgroup defined by the confounders. We believe that there is no evidence of the violation of this assumption in our study following detailed data examination. In fact, there were enough observed individuals in each subgroup defined by exposure and categorical confounders considered one at a time in our study. In addition, g-computation is less prone to this problem (relative to other methods for fitting marginal structural model using inverse probability of treatment weighting) as it relies on the outcome model specification. Fourth, we made the no interference or SUTVA (stable unit treatment value assumption)^41^. We essentially assumed here that there was little or no spillover effect. This meant, for instance, that the potential outcome under a specified exposure for a particular individual should not be affected by the potential outcome of another individual. Interference or spillover effect can occur, for example, if children of the same household were to be included in this study. In fact, if one sibling’s potential outcome under, say, a breakfast intervention (e.g., the sibling loses weight), influences another sibling’s potential outcome, for example, through motivation or modeling, there could be violation of the no interference assumption. This could also lead to standard errors being underestimated. In our study, even though sibling pairs were not included, the children belonged to the same grade level. This could also potentially lead to a violation of the no interference assumption, warranting caution in the interpretation of our results. Fifth, as is the case in all statistical modeling used for causal inference, we assumed that our models were correctly specified and that there were no other sources of bias such as from differential loss-to-follow-up, missingness not at random, sparse data, measurement error and so on.

Finally, although the current study provides a new approach to study the impact of behavioral interventions, it is important to acknowledge that the results of the study are applicable under the hypothetical scenarios that were defined in this study. As an example, the hypothetical interventions focused on meeting existing recommendations. However, it is possible for interventions to further improve the behavior of those already meeting recommendations, as dose–response relationships between some of the behaviors (e.g. PA) and body weight can exist.

## Conclusion

Using g-computation, a modern causal modeling method, the study demonstrated that a combination of dietary behavior and PA promotion and screen time reduction interventions at age 11 could have the highest beneficial effect in the reduction of BMI at age 13. Isolated behavioral interventions appeared to have a limited impact on BMI. The findings highlight the importance of a systems approach to obesity prevention focusing on multicomponent interventions.

## Data Availability

The datasets analyzed during the current study are available from the corresponding author on reasonable request.
